# Idiopathic Isolated Cilioretinal Artery Occlusion Treated with Hyperbaric Oxygen Therapy

**DOI:** 10.4274/tjo.87513

**Published:** 2016-10-17

**Authors:** Serdar Aktaş, Osman Murat Uyar, Erol Özer, Hatice Aktaş, Kadir Eltutar

**Affiliations:** 1 Dumlupınar University Faculty of Medicine, Department of Ophthalmology, Kütahya, Turkey; 2 Maltepe University Faculty of Medicine, Department of Ophthalmology, İstanbul, Turkey; 3 İstanbul Training and Research Hospital, Ophthalmology Clinic, İstanbul, Turkey; 4 Evliya Çelebi Training and Research Hospital, Ophthalmology Clinic, Kütahya, Turkey

**Keywords:** Cilioretinal artery occlusion, fundus fluorescein angiography, Hyperbaric oxygen therapy, visual field

## Abstract

Cilioretinal artery occlusion (CLRAO) is a rare event which has been reported in association with various systemic diseases. We report a case of idiopathic isolated CLRAO treated successfully with hyperbaric oxygen (HBO) therapy. A 26-year-old man presented with sudden, painless vision loss and an inferior hemivisual field defect in the left eye. Fundus fluorescein angiography revealed an occluded cilioretinal artery. After 2 weeks of HBO therapy, visual acuity improved from 20/200 to 20/20. The visual field defect improved.

## INTRODUCTION

Retinal artery occlusions (RAO) present with acute, painless loss of monocular vision. Central retinal artery occlusion (CRAO) is a rare event with an incidence of approximately 1 to 10 in 100,000.^1,2^ Symptomatic cilioretinal artery occlusion (CLRAO) is even less common; comprising about 5.3%-7.1% of all RAOs.^3,4^ To our knowledge, there have been few reports in the literature presenting isolated CLRAO treated with hyperbaric oxygen (HBO) therapy. In this study, we report a case of CLRAO treated with HBO therapy.

## CASE REPORT

**Figure 1:**
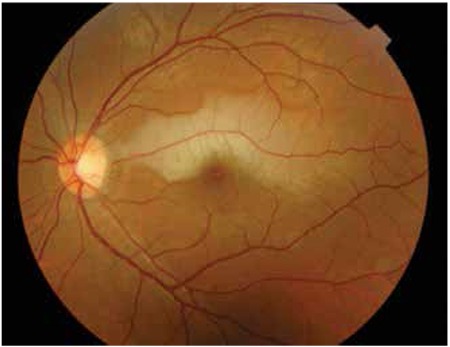
Fundus photograph at the first visit showing retinal edema adjacent to the fovea

**Figure 2:**
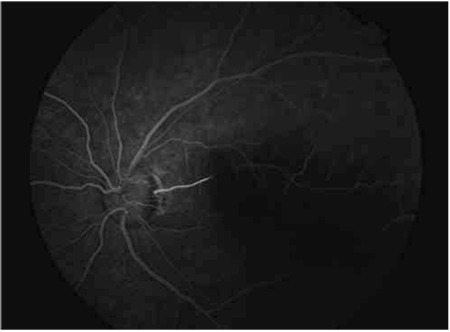
Fundus fluorescein angiography revealed an occluded cilioretinal artery

A 26-year-old man came to our clinic with a complaint of painless sudden loss of vision in the left eye, which had started 20 hours earlier. On ophthalmic examination, best-corrected visual acuity was 20/200 in the left eye and 20/20 in the right eye (Snellen chart). Intraocular pressure was 12 mmHg in both eyes. Slit-lamp examinations of anterior chambers were normal in both eyes. Funduscopy of the left eye revealed well-demarcated retinal edema centered on a cilioretinal artery adjacent to the fovea (Figure 1). We diagnosed the disease as CLRAO. After application of topical timolol+dorzolamide+bimatoprost and oral 500 mg acetazolamide, ocular massage was performed. Fundus fluorescein angiography demonstrated an occluded cilioretinal artery (Figure 2).

**Figure 3:**
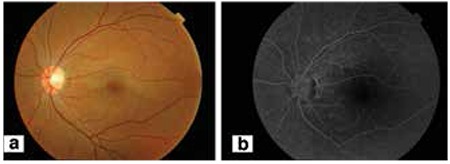
Fundus photograph (a) and fundus fluorescein angiography (b) in the second week after treatment showing recanalization of the cilioretinal artery

**Figure 4:**
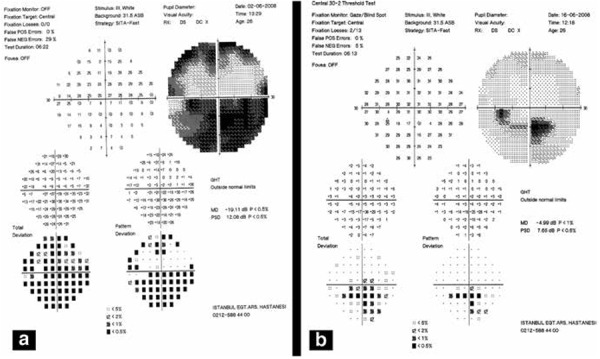
Computerized visual field testing revealed a significant decrease in the size of visual field defect after 5 sessions of hyperbaric oxygen treatment (a) and in the second week after discontinuation of treatment (b)

HBO therapy was started 22 hours after the onset of symptoms. The patient underwent 5 HBO therapy sessions (2.5 atm, 2 hours). When he returned on the third day after treatment, his visual acuity was unchanged. Five additional HBO treatments were performed, and one week later the visual acuity of the left eye had improved to 20/30. Resolution of the retinal edema was also noted. HBO treatment was discontinued after 20 sessions (total 40 hours). When he returned on the second week after treatment his visual acuity had improved to 20/20. The retinal edema was further resolved. Fundus fluorescein angiography on the second week after the end of treatment demonstrated that the cilioretinal artery was recanalized (Figure 3a, 3b). Computerized visual field testing also demonstrated a significant decrease in the size of the visual field defect (Figure 4a, 4b). His blood laboratory findings, systemic physical examination, electrocardiogram, and chest x-ray were all unremarkable. Cryoglobulin, lupus anticoagulant, and anti-cardiolipin antibodies were all negative. Antithrombin III, protein C, and protein S activities were normal.

## DISCUSSION

The central retinal artery supplies the inner retina and the surface of the optic nerve. In some individuals, the cilioretinal artery, a branch of the ciliary circulation, may supply a portion of the retina including the macula. In our patient, the cilioretinal artery entered into the retina at the optic disc margin on the temporal side, supplying some part of the upper temporal quadrant of the retina (Figure 1).

In CLRAO, vision loss results from cell death in the inner retinal layers (mainly ganglion cells) despite relative sparing of the outer layers. In order to prevent irreversible damage to the retina, HBO therapy must be provided as soon as possible after the onset of vision loss. According to the HBO treatment algorithm accepted at most centers, patients presenting within 24 hours of symptom onset should be considered for HBO therapy.^5^ Our patient received HBO therapy 22 hours after the onset of vision loss. While there are a few case reports of patients presenting after this time interval who have had positive results when treated with HBO therapy, the majority of cases do not respond when treated beyond this point.^6,7,8,9^ Hayreh et al.’s^10^ animal model, in which a CRAO induced by clamping the artery for 4 hours or longer resulted in massive and irreversible retinal damage, may not be applicable to the human situation.^11^ In the clinical setting, there are many variables, including the varying degrees and acuteness of the reduction in flow as well as the range, depending upon the patient, of differing perfusion pressures required to avoid retinal damage in different areas of the retina. In some cases of RAO, the retina, or at least a portion of the retina, may retain functional ability for a longer period of time than previously thought.^11^ Weiss^11^ reported an 81-year-old woman with mitral valve disease (on Coumadin) with a 12-day history of symptoms secondary to RAO. After 8 HBO sessions (1 hour, 1.5 atm), the visual acuity improved from counting fingers at 6 feet to 20/50 with improvement in the visual field.

There is no consensus with regard to the duration, pressure and the number of sessions of HBO treatment. Ophthalmology literature includes cases successfully treated with HBO at pressures ranging from 1.5 atm to 3 atm.^5,6,7,11^ The retina has the highest rate of oxygen consumption of any organ in the body, at 13 ml/100 g/minute.^12^ Therefore, it is very sensitive to ischemia, even more so at younger ages. Considering the young age of our patient, we preferred high pressure (2.5 atm) and long duration (2 hours) of HBO therapy. In order to be effective, the administration of supplemental oxygen must be continued until the obstructed retinal artery recanalizes, which typically occurs within the first 72 hours.^5,13^ However, in our case, the occluded cilioretinal artery was not recanalized in the first 72 hours. On the third day, after 5 HBO therapy sessions, the visual acuity was unchanged. One week after the onset of CLRAO, following a total of 10 HBO treatments, the visual acuity of the left eye improved to 20/30. Resolution of the retinal edema was also noted. HBO treatment was stopped after 20 sessions (total 40 hours). When he returned in the second week after treatment, his visual acuity had improved to 20/20. Fundus fluorescein angiography on the second week after the treatment demonstrated that the cilioretinal artery was recanalized (Figure 3).

CLRAO has been reported in association with embolism, sildenafil, systemic lupus eryhematosus, Antiphospholipid syndrome, migraine, pregnancy, systemic hypertension, and hyperhomocysteinemia.^14,15,16,17^ In our case, there was no known associated risk factor for CLRAO. To our knowledge, our patient is the first case reported as idiopathic isolated CLRAO that was treated, successfully, with HBO therapy.

We believe that HBO therapy is safe and effective in the treatment of CLRAO and should be applied until the recanalisation of the retinal artery occurs. Further research is recommended to assess the effective pressure, duration and total number of sessions of HBO therapy.

### Ethics

Informed Consent: It was taken.

Peer-review: Externally peer-reviewed.
